# Optimization of Gamma Aminobutyric Acid Production Using High Pressure Processing (HPP) by *Lactobacillus brevis* PML1

**DOI:** 10.1155/2022/8540736

**Published:** 2022-01-13

**Authors:** Atefe Ghafurian Nasab, Sayed Ali Mortazavi, Farideh Tabatabaei Yazdi, Mahboobe Sarabi Jamab

**Affiliations:** ^1^Department of Food Science and Technology, Faculty of Agriculture, Ferdowsi University of Mashhad, Mashhad, Iran; ^2^Food Biotechnology Department, Food Science and Technology Research Institute, Mashhad, Iran

## Abstract

In the present research, the production potential of gamma aminobutyric acid (GABA) using *Lactobacillus brevis* PML1 was investigated. In addition, the microorganism viability was examined in MAN, ROGOSA, and SHARPE (MRS) after undergoing high hydrostatic pressure at 100, 200, and 300 MPa for 5, 10, and 15 min. Response surface methodology (RSM) was applied to optimize the production conditions of GABA as well as the bacteria viability. Analysis of variance (ANOVA) indicated that both the independent variables (pressure and time) significantly influenced the dependent ones (GABA and bacteria viability) (*P* < 0.05). The optimum extraction conditions to maximize the production of GABA included the pressure of 300 MPa and the time of 15 min. The amount of the compound was quantified using thin-layer chromatography (TLC) and spectrophotometry. For the process optimization, a central composite design (CCD) was created using Design Expert with 5 replications at the center point, whereby the highest content of GABA was obtained to be 397.73 ppm which was confirmed by high performance liquid chromatography (HPLC). Moreover, scanning electron microscopy (SEM) was utilized to observe the morphological changes in the microorganism. The results revealed that not only did have *Lactobacillus brevis* PML1 the potential for the production of GABA under conventional conditions (control sample) but also the content of this bioactive compound could be elevated by optimizing the production parameters.

## 1. Introduction

GABA is a four-carbon nonprotein amino acid found in bacteria, plants, and vertebrate [1]. It is widely distributed in nature and plays a key role as an inhibitory neurotransmitter in the central nervous system of vertebrate [2]. This bioactive compound, which has numerous physiological activities, binds with its specific receptors located in the postsynaptic membranes of neurons. By preventing the transmittance of some neural messages, GABA is effective in suppressing stress, treating insomnia [3], restraining depression [4, 5], improving the long-term memory [6], regulating the blood pressure [7], and accelerating the protein synthesis in the brain [8]. This nonprotein amino acid can be used at certain doses in different products such as drugs, chewing gum, candy, and drinks without any side effects [9, 10]. The direct addition of chemical GABA to food products is unfavorable, because of being synthetic and unsafe, high cost, high energy consumption, and producing side effects in the product. Hence, the natural production of GABA can be introduced as an alternative way. At the same time, the amount of GABA is so negligible in plants, animals, and natural products such as cheese, green tea, rice sprouts, and brown rice. Therefore, researchers are seeking novel approaches for the mass production of GABA. For the reasonable production of this amino acid, the biological synthesis approach has attracted more attention than the other ones [11, 12].

Although numerous microorganisms can produce GABA, scientists have concentrated on the GABA-producing lactic acid bacteria (LAB) in recent years, as they are safe and, in addition to their applications in the food industry, they have specific physiological activities which can be compatible with the environment [13, 14]. Moreover, the presence of glutamic acid decarboxylase (GAD) in some LAB has raised their importance in GABA production [15]. GAD is a unique enzyme which catalyzes the separation of the carboxyl group from glutamate and converts it into GABA. This enzyme brings about the irreversible conversion of L-glutamate into GABA [16]. Among the GABA-producing LAB, Lactobacillus can be used for GABA production, among which *Lactobacillus brevis* is one the major ones. It is a safe Gram-positive species which is widely used as a probiotic strain in the production of fermented foods [17, 18].

Considering the information presented and the importance of the production of the nonprotein compound of GABA in the food and pharmaceutical industries, discovering an effective treatment which can give rise to the manufacture of this substance is of great importance whether in the food industry or in the medical and pharmaceutical sciences [19].

Nowadays, the rise in consumers' demand for high-quality food products has had the food industry pay attention to nonthermal technologies [19, 20]. High pressure processing (HPP) or high hydrostatic pressure (HHP) is one of the most significant nonthermal methods for extending the shelf-life of food products, due to the inactivation of microorganisms [21, 22]. At the same time, HHP is not limited to pasteurization and sterilization. It is also applied to extraction purposes (particularly the extraction of bioactive compounds) and acceleration of the expulsion of intracellular components [23, 24]. In case of utilizing the pressures lower than the lethal limit, HHP can cause stress on the microorganism and alter its metabolite secretion, thus improving the extraction efficiency of the metabolites [25, 26].

RSM is a series of mathematical and empirical techniques useful for investigating the effects of some variables on the performance and quality of a process or product with the aim of the modeling and optimization of complicated processes. RSM is vastly employed owing to its high optimization accuracy as well as cutting down on experimentation costs. This method is used to examine the main, quadratic, and interactive effects of independent variables on responses.

The aim of this study, as a novel research, is to optimize the conditions of GABA extraction from the probiotic strain of *Lactobacillus brevis* PML1 through RSM and achieve the highest yield of GABA production.

## 2. Materials and Methods

### 2.1. Microorganism Activation

*Lactobacillus brevis* PML1, isolated from Tarkhineh, was supplied from the microbial collection of the Department of Food Science and Technology, Faculty of Agriculture, Ferdowsi University of Mashhad. MRS agar and MRS broth (Merck Co., Germany) were utilized for the activation of the microorganism [7].

### 2.2. Sample Preparation

For activation, the lyophilized bacteria were transferred to the sterile MRS broth comprised of agar12 g/l, diammonium hydrogen citrate 2 g/l, yeast extract 5 g/l, D-glucose 20 g/l, dipotassium hydrogen phosphate 2 g/l, sodium acetate 5 g/l, magnesium sulfate 0.1 g/l, meat extract 5 g/l, universal peptone 10 g/l, and manganese sulfate 0.05 g/l. Subsequently, the bacteria were incubated at 30°C for 24 h [7]. Afterwards, a microbial suspension was prepared with an absorbance value of 0.08 at 600 nm (equivalent to 0.5 McFarland).

### 2.3. High Hydrostatic Pressure and GABA Production

In order to exert pressure on the microorganism and increase the GABA production yield, a HHP machine (Research Institute of Food Science and Technology, Mashhad, Iran) was employed, operating at pressures up to 600 MPa. After preliminary experiments, the pressure levels of 100, 200, and 300 MPa and durations of 5, 10, and 15 min were chosen. It should be noted that the exerted pressures were considered below the lethal limit of the microorganism. For the process optimization, a CCD was created using Design Expert (v 7.0.0) with 5 replications at the center point (Table 1). In order to exert the HHP, the prepared microbial suspension (equivalent to 0.5 McFarland) was poured into 1.5 ml microtubes and exposed to the different treatments. An unpressurized sample was considered to be the control.

In order to investigate the effect of the HHP on the GABA production by *Lactobacillus brevis* as well as verifying the viability of the bacteria, after exerting the HHP, 1 ml of each microbial suspension was transferred to the falcons containing 5 ml of sterile MRS broth and incubated at 30°C for 72 h [11, 27]. The control also underwent the same procedure without exerting the HHP.

### 2.4. Viability of Pressurized Cells

In order to examine the effect of HHP on the bacterial count, the total count of the microorganism was performed using MRS agar. To that end, serial dilutions were prepared from the microbial suspensions before and after exerting the HHP and also after 72 h of incubation. After culturing on MRS agar, the plates were incubated at 37°C for 48 h. Finally, the number of the formed colonies was counted using a colony counter [28, 29].

### 2.5. Evaluation of GABA Production

#### 2.5.1. Examination of GABA Production Using TLC and Spectrophotometry

Given that GABA is a metabolite which is secreted out of the microbial cell, the supernatant was used after removing the bacterial pellet to determine the amount the GABA produced. For this purpose, after 72 h of incubation, the microbial suspension was centrifuged at 861 g for 10 min. After that, the resulting supernatant was filtered through a 0.22 *μ*m filter to remove the probable microorganisms. TLC was applied to assess the GABA content. To that end, a horizontal line was drawn with a pencil at a 2 cm distance from the bottom of the active silica gel plate (60 F254). Dotting was done at 1 cm intervals on the line. Subsequently, using a capillary tube, 2 *μ*l of each sample (pure GABA, the control and the supernatant containing GABA) was taken and spotted on the dots. After the dots were dried, the plate was placed in the solvent (distilled water: butanol: acetic acid (2 ml : 5 ml : 2 ml)). After rising the solvent off the plate up to a length of about 8 cm, the ninhydrin solution (ninhydrin: butanol: acetic acid (0.75 g : 50 ml : 1.5 ml)) was sprayed onto the plate which was then put in an oven at 100°C for 80 min. After the emergence of the bands, the ones facing GABA were cut and separately dissolved in the solution of ethanol 75% and copper sulfate 0.6% (38 ml : 2 ml). Next, the mixture was put in a shaking incubator (50 g) at 40°C for 60 min. After that, the absorbance value of each sample was measured at 512 nm using a spectrophotometer (UV 1800 Shimadzu, Japan). Afterwards, the GABA content of each sample was quantified using the obtained equation. In order to draw the standard curve and achieve the respective equation, the pure GABA was prepared at different concentrations (100, 200, 400, 800, and 1000 ppm), dotting was carried out on the TLC plate, and the absorbance value of each dot was measured spectrophotometrically as described above. By applying the absorbance values to the standard curve, the following equation (*y* = 1322.9*x* + 25.867) was obtained with a determination coefficient (*R*^2^) of 0.96 [30].

#### 2.5.2. Confirmation of GABA Content by HPLC

After analyzing the obtained data and optimizing the process through RSM, the GABA content of the optimum sample was quantified using HPLC to verify the results of TLC and spectrophotometry [1]. The optimal sample was centrifuged at 861 g for 15 min, and the supernatant was passed through a 0.22 *μ*m filter. The filtrate was mixed with sodium bicarbonate 0.2 mM at a ratio of 9 : 1 and pH = 9.8. After the addition of the derivatizer of dansyl chloride at a concentration of 8 g/l, the sample was kept in the dark at 30°C. After the preparation and derivatization of the GABA standards at 150, 350, and 750 mg/l, HPLC was conducted using column 18 and UV detector at 254 nm [8]. In order to accomplish the standard curve and the linear regression equation, the three internal standards of GABA (150, 350, and 750 ppm) were injected into the column, and the following equation (*y* = 0.4907*x* + 2.4747) was achieved with an *R*^2^ of 0.99 [9].

### 2.6. Investigation of the Structural Changes in the Bacteria after HHP Using SEM

In order to verify the impacts of HHP on the structure of the bacterial cells and observe the morphological changes in the control and optimum samples, they were firstly centrifuged. After the obtained pellets were dried, they were immobilized with glutaraldehyde 2.5% (Sigma-Aldrich, USA) in phosphate buffer 0.1 M (pH = 7.4) at room temperature for 1 h. Next, the samples were rinsed twice with the phosphate buffer. For the secondary immobilization, they were mixed with osmium tetroxide 2% (Proxytech, Australia) in the same buffer and temperature for 30 min. After rinsing with the buffer for three times, the samples were dried with ethanol at concentrations of 30, 50, 70, 80, and 100 (*v*/*v*) and coated with a layer of gold. Eventually, they were observed with a scanning electron microscope (LEO-Germany, VP-1450) [31].

## 3. Results and Discussion

### 3.1. Assessment of GABA Production (TLC)

**Figure 1:**
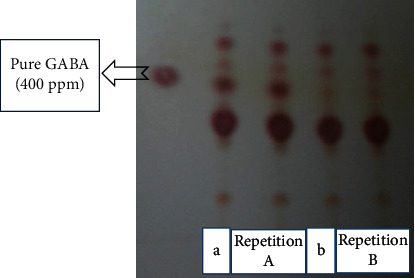
Thin-layer chromatography to evaluate the production of GABA in MRS broth: (a) pressure of 300 MPa and time of 15 min; (b) pressure of 100 MPa and time of 5 min.

There are a number of methods for the primary detection of GABA; however, they are not considered to be desirable because of being expensive and time-consuming. TLC is suitable for the simultaneous examination of a large number of samples. At the same time, it does not require expensive equipment, thus being recommended for the determination of GABA content. Many researchers have employed TLC for the primary screening of GABA production (Figure 1).

### 3.2. Optimization

In order to determine the trend of the variations in GABA production, a model should be selected, which best fits the experimental data and properly explains the effects of the independent variables on GABA production. The probability of the lack of fit of the quadratic model was equal to 0.07 in this research. Consequently, this model could appropriately analyze and fit the empirical data and chosen to describe the variations in the responses (Table 2). In order to determine the model accuracy, *R*^2^ and the lack of fit test were employed. *R*^2^ was equal to 0.92; as a result, this model had a good prediction ability to investigate the GABA production [32].

ANOVA is necessary for precisely studying the effects of each factor on GABA production and validating the obtained data. According to ANOVA, *P* < 0.05 revealed that the quadratic model was suitable for the explanation of the effects of the factors on GABA production (Table 3) [32].

### 3.3. Effects of Pressure and Time on GABA Production

**Figure 2:**
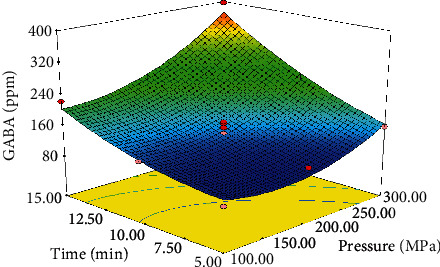
Interactive effect of pressure and time on GABA production by *Lactobacillus brevis*.

HHP is a nonthermal technology currently applied to increase food safety through bacteria destruction [33, 34] and extend the quality and shelf-life of food products without undesirably impacting on their odor, taste, and texture. In the present research, HHP was used to elevate the GABA production yield by *Lactobacillus brevis*. Based on the literature, exerting high HHPs can produce destructive (lethal) effects on microorganisms including LAB. For instance, Malone et al. [35] verified the effects of different pressures on *Lactococcus lactis ssp. lactis* at 25°C for 5 min. The results showed that the bacteria were completely inactivated at 400-800 MPa. According to preliminary experiments, the pressures lower than the lethal limit were taken into account in the present research. The results of the preliminary tests (data not shown) indicated that *Lactobacillus brevis* was destructed at pressures exceeding 400 MPa or durations longer than 15 min. Therefore, the pressures lower than the lethal limit were chosen and examined at different times. The process time is another key factor remarkably affecting the GABA content. It can be observed in Figure 1 that the GABA content increased as the process time was elevated from 5 to 15 min. This could be due to the rise in the number of the bacteria during fermentation in addition to the compression of the bacteria caused by HHP. The results also demonstrated that the maximum GABA content was found to be 397.73 ppm which was obtained under the optimum conditions of 300 MPa and 15 min (Figure 2).

With an increase in HHP from 100 to 300 MPa, the GABA content followed an increasing trend, conforming to the results previously obtained by [3, 36]. This also applies to the process time. The highest GABA content was associated with the conditions of 300 MPa and 15 min.

Ferreira et al. [37] studied the effect of pressure at three levels on *Saccharomyces cerevisiae* to raise its bioethanol production efficiency. They exerted 15, 25, and 35 MPa on the yeast at 30°C for 72 h and observed that ethanol was most produced at 25 MPa. Consequently, it can be deduced that low pressures at short times cannot be that efficient, and pressure and time highly depend on one another. Corrales et al. (2009) investigated the effect of HHP on the amount of the anthocyanin extracted from grape skin. They applied 200, 400, and 600 MPa. Using HPLC, the highest anthocyanin content was determined at 600 MPa. These researchers also stated that HHP was not restricted to pasteurization and sterilization and could also be used for extraction purposes (in particular the extraction of bioactive compounds).

### 3.4. Effect of Pressure and Time on Bacterial Cell Viability

**Figure 3:**
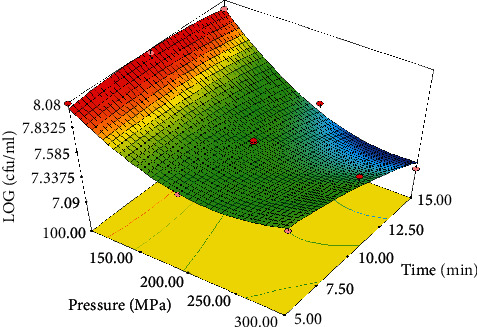
Interactive effect of pressure and time on viability of *Lactobacillus brevis*.

Total count of the microorganisms was carried out using MRS agar to verify the influence of HHP on the bacterial count of the control and the treated samples before and after exerting the HHP, as well as after 72 h of incubation. The results showed that the growth of *Lactobacillus brevis* was equal to 8.2 cfu/ml in the control after being cultured in MRS agar and incubated for 72 h. After exerting the HHP, the growth varied from 7.09 to 8.07 logarithmic cycles.  Figure 3 illustrates the interactive effect of time and pressure on the bacteria viability. As the pressured increased from 100 to 300 MPa and the time rose from 5 to 15 min, the number of the bacterial cells was lowered significantly (*P* < 0.05); however, only a reduction of one logarithmic cycle was observed, which is in agreement with the findings obtained by Mok et al. (2006) who studied the impact of HHP on the LAB present in red wine. They utilized HHP at 100-150 MPa for 0-30 min and stored the wine samples for 14 days after the treatments. These researchers declared that the bacterial population declined beyond the permitted limit after the treatment of the wine at 300 MPa for 20 min or 350 MPa for 5 min.

Based on the obtained results, with a rise in HHP and the process time, it will be possible to provide suitable conditions for increasing GABA production by *Lactobacillus brevis*. Therefore, HHP can be applied as a nonthermal technology and improve the ability of the bacteria to produce more GABA. Nevertheless, high pressures can destruct the microorganism. Furthermore, the HHP exertion time is another major factor in GABA production, as the bacterial population decreases to below the permitted level with an increase in the HHP exertion time [38, 39].

### 3.5. Optimization and Model Validation Using HPLC

The results of optimization demonstrated that the GABA content of the control was equal to 37.11 ppm with an absorbance value of 0.0085. On the other hand, the largest amount of GABA (397.73 ppm) was produced under the conditions of 300 MPa and 15 min which were determined as the optimum conditions by the RSM optimization algorithm with a desirability of 0.91, considering the maximization of the GABA content and the viability of the bacterial cells being in range.

**Figure 4:**
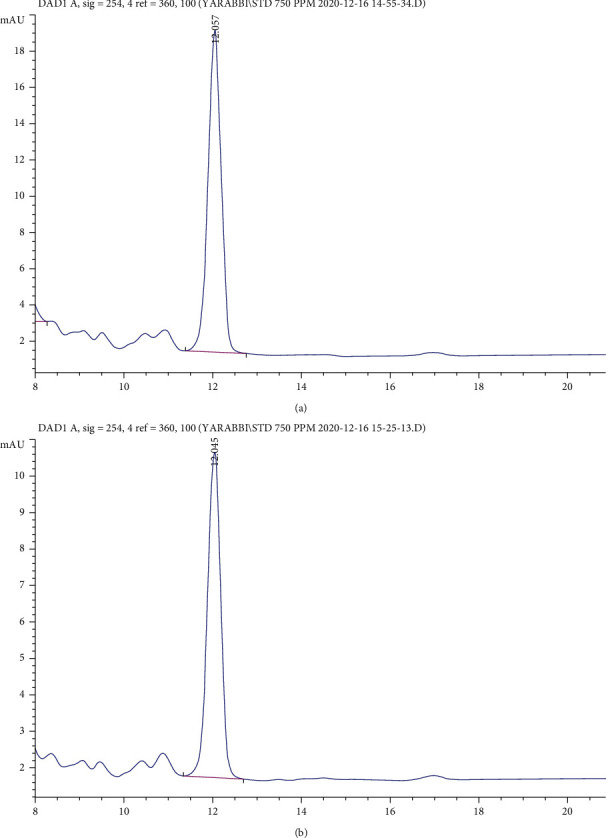
Chromatograms of (a) optimum sample and (b) pure GABA standard at 750 ppm.

A confirmation test was taken into consideration to validate the model. In this test, the GABA content was measured again under laboratory conditions and compared with the GABA content predicted by the model using HPLC. Given the injection of the GABA internal standards, the average retention time was equal to 12 s for GABA, and the area under the curve of the optimum sample was reported to be 192.67. Using the obtained equation, the GABA content of the optimum sample was estimated to be 388.15 ppm. It was revealed that the results predicted by the model highly conformed to the empirical ones. It should be mentioned that the unknown peaks in the chromatogram of the optimum sample are related to the compounds used for the sample derivatization (Figure 4).

Chunlong et al. (2013) examined the effects of two different conditions (pH = 5 at 35°C and pH = 4.5 at 40°C) on the content of the GABA produced by *Lactobacillus brevis* CGMCC 1306. They quantified the GABA content through HPLC and reported it to be 474.79 mmol/l at pH = 4.5 and 40°C. Park and Oh (2007) also optimized the conditions for *Lactobacillus brevis* OPY-1, isolated from yogurt, and measured the GABA content 2.5 g/l by HPLC.

### 3.6. Investigation of the Microorganism Morphology by SEM

In order to investigate the impact of HHP on the bacterial cell morphology, the control and optimum (300 MPa for 15 min) samples were observed through SEM.

**Figure 5:**
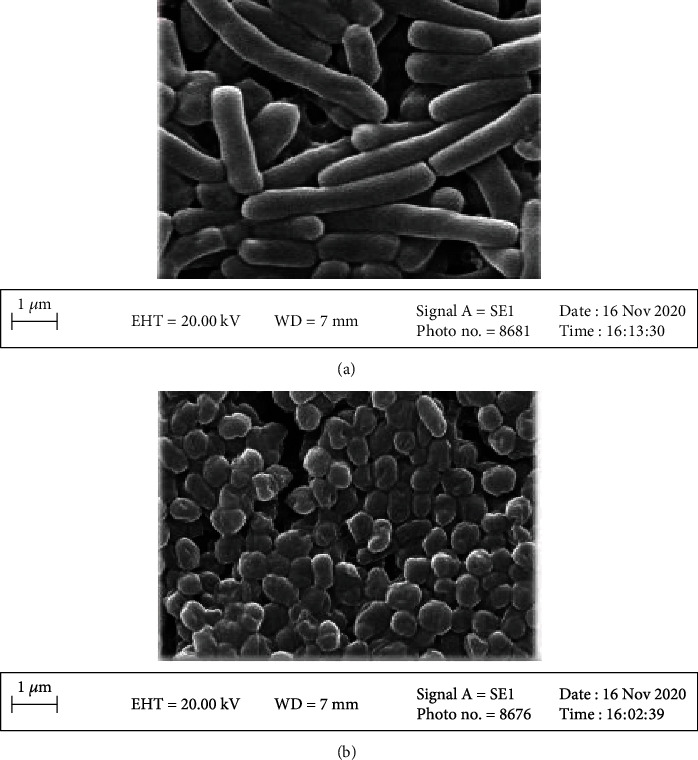
*Lactobacillus brevis* morphology (a) control sample and (b) optimum sample.

As can be seen in Figure 5, the rod-shaped cells of *Lactobacillus brevis* which had been pressurized at 300 MPa for 15 min were compressed compared with the control cells. This deformation could be ascribed to the effect of the protective agent of the cell against HHP. It can be deduced that HHP, at pressures lower than the lethal limit, can raise the GABA production yield without damaging the bacterial cell and killing the microorganism [40].

## 4. Conclusions

The present research indicated that HHP brought about approximately one thousand percent increase in the GABA content of the optimum sample, compared with the control. Therefore, the biological synthesis of GABA can be enhanced with the help of this technology and exerting the pressures lower than the lethal limit (pressure levels of 100, 200, and 300 MPa and durations of 5, 10, and 15 min were chosen. It should be noted that the exerted pressures were considered below the lethal limit of the microorganism), thus amazingly developing the food and pharmaceutical industries. Moreover, investigation into the effects of HHP on the extraction efficiency of other bioactive compounds is recommended for further studies.

## Figures and Tables

**Table 1:** Amount of GABA produced in each sample (ppm) and investigation of bacteria logarithmic growth.

Number	HHP (MPa)	Time (min)	Absorbance value	GABA (ppm)	(Log cfu/ml)
1	100	5	0.0462	86.98	8.07
2	100	10	0.0775	128.39	8.04
3	100	15	0.1491	223.11	8.0
4	200	5	0.0661	113.31	7.6
5	200	10	0.0864	140.21	7.47
6	200	10	0.1090	170.11	7.5
7	200	10	0.0977	155.14	7.49
8	200	10	0.1056	165.62	7.54
9	200	10	0.0790	130.44	7.56
10	200	15	0.1318	200.23	7.40
11	300	5	0.0999	158.02	7.69
12	300	10	0.1531	228.4	7.6
13	300	15	0.2811	397.73	7.09

**Table 2:** Analysis of variance for GABA production.

Analysis of variance table (partial sum of squares-type 3)						
Source	Sum of squares	DF	Mean square	*F*-value	*P* value Prob > *F*	
Model	67608.80	5	13521.76	17.27	0.0008	Significant
A-pressure	19914.62	1	19914.62	25.44	0.0015	
B-time	35691.14	1	35691.14	45.60	0.0003	
AB	2682.20	1	2682.20	3.43	0.1066	
A^2^	4944.47	1	4944.47	6.32	0.0402	
B^2^	1181.87	1	1181.87	1.51	0.2589	
Residual	5479.18	7	782.74			
Lack of fit	4352.46	3	1450.82	5.15	0.0736	Not significant

**Table 3:** Analysis of variance for *Lactobacillus brevis* viability.

Analysis of variance table (partial sum of squares-type3)						
Source	Sum of squares	DF	Mean square	*F*-value	*P* value Prob > *F*	
Model	0.89	5	0.18	39.13	<0.0001	Significant
A-pressure	0.50	1	0.50	109.62	<0.0001	
B-time	0.13	1	0.13	27.72	0.0012	
AB	0.070	1	0.07	15.43	0.0057	
A^2^	0.19	1	0.19	41.63	0.0003	
B^2^	0.009	1	0.009	2.05	0.1954	
Residual	0.032	7	0.004			
Lack of fit	0.026	3	0.008	6.42	0.0522	Not significant

## Data Availability

All data generated or analyzed during this study are included in this published article.
